# Phosphorylation of Dynamin-Related Protein 1 (DRP1) Regulates Mitochondrial Dynamics and Skeletal Muscle Wasting in Cancer Cachexia

**DOI:** 10.3389/fcell.2021.673618

**Published:** 2021-08-05

**Authors:** Xiangyu Mao, Yihua Gu, Xiangyu Sui, Lei Shen, Jun Han, Haiyu Wang, Qiulei Xi, Qiulin Zhuang, Qingyang Meng, Guohao Wu

**Affiliations:** ^1^Department of General Surgery/Shanghai Clinical Nutrition Research Center, Zhongshan Hospital, Fudan University, Shanghai, China; ^2^Shanghai Institute of Planned Parenthood Research, Shanghai, China

**Keywords:** dynamin-related protein 1, cancer-associated cachexia, mitochondria fission, skeletal muscle, atrophy

## Abstract

**Background:**

Cancer-associated cachexia (CAC) is a syndrome characterized by skeletal muscle atrophy, and the underlying mechanisms are still unclear. Recent research studies have shed light on a noteworthy link between mitochondrial dynamics and muscle physiology. In the present study, we investigate the role of dynamin-related protein 1 (DRP1), a pivotal factor of mitochondrial dynamics, in myotube atrophy during cancer-associated cachexia.

**Methods:**

Seventy-six surgical patients, including gastrointestinal tumor and benign disease, were enrolled in the study and divided to three groups: control, non-cachexia, and cancer-associated cachexia. Demographic data were collected. Their rectus abdominis samples were acquired intraoperatively. Muscle fiber size, markers of ubiquitin proteasome system (UPS), mitochondrial ultrastructure, and markers of mitochondrial function and dynamics were assayed. A cachexia model *in vitro* was established *via* coculturing a C2C12 myotube with media from C26 colon cancer cells. A specific DRP1 inhibitor, Mdivi-1, and a lentivirus of DRP1 knockdown/overexpression were used to regulate the expression of DRP1. Muscle diameter, mitochondrial morphology, mass, reactive oxygen species (ROS), membrane potential, and markers of UPS, mitochondrial function, and dynamics were determined.

**Results:**

Patients of cachexia suffered from a conspicuous worsened nutrition status and muscle loss compared to patients of other groups. Severe mitochondrial swelling and enlarged area were observed, and partial alterations in mitochondrial function were found in muscle. Analysis of mitochondrial dynamics indicated an upregulation of phosphorylated DRP1 at the ser616 site. *In vitro*, cancer media resulted in the atrophy of myotube. This was accompanied with a prominent unbalance of mitochondrial dynamics, as well as enhanced mitochondrial ROS and decreased mitochondrial function and membrane potential. However, certain concentrations of Mdivi-1 and DRP1 knockdown rebalanced the mitochondrial dynamics, mitigating this negative phenotype caused by cachexia. Moreover, overexpression of DRP1 aggravated these phenomena.

**Conclusion:**

In clinical patients, cachexia induces abnormal mitochondrial changes and possible fission activation for the atrophied muscle. Our cachexia model *in vitro* further demonstrates that unbalanced mitochondrial dynamics contributes to this atrophy and mitochondrial impairment, and rebuilding the balance by regulating of DRP1 could ameliorate these alterations.

## Introduction

Cancer-associated cachexia is a multifactorial system syndrome characterized by loss of muscle mass, with or without loss of fat mass (Baracos et al., 2018). Patients diagnosed with cancers from the digestive system (such as pancreatic, colorectal, gastro-esophageal) have a relatively high rate (45–70%) of developing cachexia at the end of life (Amano et al., 2017). Muscle wasting and accompanied dysfunction markedly reduce their life qualities and survivals (Penna et al., 2019). Therefore, numerous research studies have been looking for therapies to prevent muscle loss while specific treatments remain rare (Martin and Freyssenet, 2021). The mitochondrion is a highly dynamic organelle that undergoes fuse and division under physiological conditions (Chan, 2012). Fusion results in elongated mitochondria and an expanded network. This machinery dilutes the damaged material from impaired mitochondria into the healthy network, avoiding the accumulation of dysfunctional mitochondria and thus helping to maintain overall function (Twig et al., 2008). In contrast, fission divides damaged or dysfunctional components from this network, and these shorter mitochondria shall be removed by mitochondrial autophagy (mitophagy) further. Therefore, the balance of the fusion and fission systems is crucial to maintaining mitochondrial physiology (Westermann, 2010). On the other hand, these two machineries are manipulated by some crucial factors. Fusion of the outer mitochondrial membrane (OMM) is controlled mainly by the GTPases mitofusin 1 (MFN1) and mitofusin 2 (MFN2), located at OMM (Chen et al., 2003). For the inner mitochondrial membrane (IMM), optic atrophy protein 1 (OPA1), an IMM membrane-bound dynamin-like GTPase, is required (Ban et al., 2017). Mitochondrial fission is dependent primarily on the cytosolic GTPase, DRP1. These proteins are recruited to the marked division sites and bind to the OMM adaptors, such as mitochondrial fission 1 (Fis1), mitochondrial fission factor (MFF), and mitochondrial elongation factor 2/mitochondrial dynamics protein 49 (MiEF2/MiD49). Then, the bindings facilitate the oligomerization of DRP1, forming a ring-like structure to mediate scission of mitochondrial membranes (Kraus and Ryan, 2017).

Recently, research studies have uncovered the intimate relationship between mitochondrial dynamics and muscle maintenance. Alterations in mitochondrial distribution, morphology, and function are observed in atrophic muscles of aging, muscle disuse, chronic obstructive pulmonary disease (COPD), and different neuromuscular disorders. Additionally, this is also evident in some preclinical models of cachexia (Romanello and Sandri, 2021). Yet, clinical studies on mitochondrial metabolism, including dynamics and function, are scarce and required (Dolly et al., 2020). In this study, we aim to investigate the mitochondrial dynamics and function in clinical patients of cachexia. We noticed that mitochondria in atrophic muscles of patients with gastrointestinal cachexia were enlarged and morphologically abnormal. Moreover, a change in mitochondrial function was observed. The phosphorylation activity of DRP1 was induced, suggesting active fission. Further, we established a model *in vitro* to explore the character DRP1 plays during this process. The regulation of balance of mitochondrial dynamics with a DRP1 inhibitor, Mdivi-1, could alleviate the atrophy in C2C12 myotube induced by cachexia circumstance. This is due to its effect on mitigating protein breakdown, restoring impaired mitochondrial function and MMP, and abrogating the production of mitoROS. Knockdown and overexpression of DRP1 in myotube further verified our assumption. Taken together, these phenomena shed new light on the importance of mitochondrial dynamic balance in cachetic atrophy, which was dominated by DRP1. The corresponding targets may provide reference for future therapy.

## Materials and Methods

### Clinical Data Collection

A total of 76 patients were enrolled in this study and divided into three groups: control (C; *n* = 20), non-cachexia (NC; *n* = 27), and cancer-associated cachexia (CAC; *n* = 29). Patients were diagnosed with malignant tumor or benign diseases (such as hernia) for the first time and had not received radiotherapy, chemotherapy, or targeted therapy before operation. Cancer cachexia was defined as weight loss > 5% within the recent 6 months (Fearon et al., 2011; Arends et al., 2017). Abdominal computed tomography examination was performed within 2 weeks preoperation. Demographic and clinical data including age, weight loss, body mass index (BMI), biceps circumference, waist circumference, and nutritional biomarkers were collected. This project conformed to the rules of the Declaration of Helsinki and was approved by the Ethics Committee of Zhongshan Hospital. Written informed consent for the study procedures was obtained from the patients. Muscle samples were collected from the rectus abdominis during operation and stored in 4% paraformaldehyde, fixation liquid for electron microscopy, or liquid nitrogen respectively for different purposes. Tumor volume was calculated using the Jones volume formula *V* = 1/2 (Length × Width^2^) (Naito et al., 1986). The TNM stage is determined according to the latest NCCN guidelines.

### CT Scan

CT images were analyzed using ImageJ software (1.5.2; National Institutes of Health, Bethesda, MD, United States) as previously described (Gomez-Perez et al., 2016). The specific Hounsfield unit (HU) threshold for skeletal muscle (−29 to +150) was set. The slice across the third lumbar vertebra (L3) was extracted. Then, muscle areas were measured and adjusted with height (cm^2^/m^2^: muscle area/height^∗^height) to transform to the skeletal muscle index (SMI), an indicator that correlates well with the skeletal muscle mass of the whole body (Prado et al., 2008). The average HU of L3 skeletal muscle was assessed to reflect muscle composition as skeletal muscle density (SMD). A low density means elevated intramuscular lipid mass that contributes to muscle weakness (Goodpaster et al., 2001).

### Measurement of CSA

To determine the cross-sectional area (CSA) of the rectus abdominis, muscle was stained with hematoxylin and eosin to observe the boundary of each fiber. ImageJ software (1.5.2; National Institutes of Health, Bethesda, MD, United States) was utilized to calculate the average CSA. For each sample, more than 40 fibers (per image) were analyzed in four to six randomly selected images (*n* = 5 for the NC and CAC groups; *n* = 3 for the C group).

### Transmission Electron Microscope Scan

Samples (volume of approximate 1 mm^3^) were placed into 4°C precooled fixation solution (2% paraformaldehyde, 2.5% glutaraldehyde) and preserved for at least 24 h. After rinsing, they were postfixed with 1% osmium tetroxide at 4°C. Dehydrating by acetone and infiltrating with embedding medium for 48 h, the samples were polymerized at 60°C for 48 h and cut into slices for observation. For each sample, calculations of mitochondrial number and area were performed in four to six randomly selected images (96.9 μm^2^ per image) (*n* = 3), using ImageJ software (1.5.2; National Institutes of Health, Bethesda, MD, United States).

### Cell Culture

All cells grew in high-glucose Dulbecco’s modified Eagle medium (DMEM) with 100 units/ml penicillin and 100 μg/ml streptomycin (KeyGen Biotech, Jiangsu, China) containing 10% fetal bovine serum (FBS) (CellSera, Rutherford, NSW, Australia). C2C12 myoblasts were cultured until reaching at least 95% confluence and then removed to DMEM with 2% horse serum (HS) (Gibco, Grand Island, NY, United States) to differentiate for 4 days.

### Conditional Medium Collection

C2C12 myoblast is an eternal mouse cell strain originated from fast-twitch muscle. Under the condition of low or no serum, they would fusion and differentiate into myotubes, which is widely used among muscle research studies *in vitro* (Yaffe and Saxel, 1977). C26 colon cancer cells secrete a number of cellular factors, including IL-6, TNF-α, IL-1β, leukemia inhibitory factor (LIF), ciliary neurotrophic factor (CNTF), and interferon-γ (IFN-γ). These factors contribute to the progress of cachexia. Therefore, it has been recognized as a preclinical model to treat myotubes with medium from C26 cells, to mimic cachexia conditions (Argiles et al., 2003; McLean et al., 2014; Seto et al., 2015). C26 conditional medium (C26 CM) was acquired using the method of Seto (Seto et al., 2015). Briefly, C26 cells were cultured in DMEM with 10% FBS until reaching 90% confluence. Then the medium was replaced with DMEM with 2% HS. CM was collected 24 h later and centrifuged at 1,000 G for 5 min. The supernatant was carefully collected as stock solution and diluted to 33% using DMEM with 2% HS as working solution. The control group was treated with DMEM with 2% HS.

### Construction of Lentiviral Vectors Modulating DRP1 Expression and Cellular Transduction

To more deeply understand the role DRP1 plays, we delegated GeneChem Inc. (Shanghai, China) to construct lentiviruses containing the small interfering RNA (siRNA) targeting DRP1 and the DRP1 plasmid, respectively. Taking siRNA as an example, the brief protocol was as follows: firstly, three siRNAs of target sequence and a control siRNA were designed. After synthesis of polymerase chain reaction (PCR) products based on these designs, they were cloned into the central part of a GV248 (11.5 kb) lentiviral vector. GV248 was the vector expressing the correlated siRNA, enhanced green fluorescent protein (eGFP), and puromycin resistance gene. Afterward, the vectors were transformed into Escherichia coli (DH5α) and the positive transformants were identified by PCR. X-tremeGENE reagent (Roche, Basel, Switzerland) was used to transfect 293T cells with the plasmid mixtures (GV248 with auxiliary packaging plasmids Helper 1.0 and Helper 2.0) for 48 h. Then, lentiviruses were harvested *via* collection of the supernatants, followed by concentrations and viral titer measurement. Furthermore, a GV358 lentiviral vector with a DRP1 expression plasmid was constructed with a similar protocol.

As for the process of transduction, myoblasts were firstly infected with lentiviruses at a multiplicity of infection (MOI) of 100 for 12 h. After culture for 72–96 h, we evaluated the efficiency of transduction by observing the fluorescence intensity of eGFP under fluorescence microscopy (Ti-E + A1R + STORM; Nikon, Tokyo, Japan). Cell lines with a transduction ratio over 80% were used. Then, the expression of DRP1 was validated by Western blotting. In order to build a cell line with a stable transduction, puromycin selection (concentrations of 0.1/0.3/0.5/0.7/0.9/1.1/1.3/1.5/1.7/1.9 μg/ml) was performed and 0.9 μg/ml was chosen as optimal.

### Measurement of Myotube Diameter

Myotubes were visualized and photographed under a biological microscope (Nikon ECLIPSE TS100-F, Japan). ImageJ software (1.5.2; National Institutes of Health, Bethesda, MD, United States) was used to determine the diameter of myotubes. The average diameter per myotube was calculated by three different points distributed along the whole myotube. For each group, more than 30 myotubes were randomly selected to form six to eight fields. Alternatively, myotubes transfected with lentivirus were stained with an anti-MHC antibody, MF20 (Developmental Studies Hybridoma Bank, University of Iowa, United States), overnight at 4°C and then incubated with second antibody Alexa Flour 488 (Thermo Fisher Scientific, Waltham, MA, United States) for 1 h. These myotubes were visualized by a confocal immunofluorescence microscope (Ti-E + A1R + STORM; Nikon, Tokyo, Japan).

### Quantitative Real-Time PCR

Total RNA from muscles was isolated using TRIzol Reagent (Thermo Fisher Scientific, Waltham, MA, United States), following the manufacturer’s instructions. Two micrograms of RNA was used for reverse transcription with the high-capacity cDNA reverse transcription kit (TianGen, Shanghai, China). Quantitative real-time PCR was performed using a FastStart Essential DNA Green Master (Roche, Basel, Switzerland) in the 7900HT PCR system (Applied Biosystems, Foster City, CA, United States). HSP90AB1 and GAPDH were used as endogenous control for human and cell samples, respectively. Moreover, specific primers are shown in Supplementary Table 1. The 2^–Δ^^Δ^^CT^ method was used for analysis of the relative expression of genes in SDS RQ Study software (Applied Biosystems, Foster City, CA, United States).

### Western Blot

For muscle tissue, a sample of 50–100 mg was cut into pieces and lysed in radio immunoprecipitation assay (RIPA) lysis buffer (#WB0101, Biotech WELL, Shanghai, China) using a glass homogenizer. For cells, they were harvested and lysed in RIPA for 15 min. These processes were performed on ice. Then, they were centrifuged at 12,000 G for 30 min. Furthermore, the supernatants were collected and boiled with SDS loading buffer. Proteins were separated by SDS-PAGE electrophoresis and transferred to NC membranes. After blocking for 1 h with 5% milk, membranes were incubated with a corresponding primary antibody overnight at 4°C and then incubated with a corresponding secondary antibody for 1 h. Finally, the ECL image system (Tanon 5200s, Shanghai, China) was used for analysis. The primary antibodies were phosphorylated-DRP1(ser616) (Absin, #137991), phosphorylated-DRP1(ser637) (CST, #6319), DRP1 (Sab, # 40853), FIS1 (Proteintech, #10956-1-AP), MFF (Proteintech, #17090-1-AP), MFN1 (Proteintech, #13798-1-AP), MFN2 (Proteintech, #12186-1-AP), OPA1 (Proteintech, #27733-1-AP), MuRF-1 (Proteintech, #55456-1-AP), Atrogin-1 (Proteintech, #67172-1-Ig), COX IV (Proteintech, #11242-1-AP), Cyto C (Proteintech, #10993-1-AP), TOM20 (Proteintech, #11802-1-AP), TIM44 (Proteintech, #13859-1-AP), PRX3 (Proteintech, #10664-1-AP), MyoD (Proteintech, #18943-1-AP), MyoG (Proteintech, #67082-1-Ig), GAPDH (ABclonal, #AC001), and Complex I (Proteintech, #14794-1-AP).

### Mitochondrial Morphology and Mass

Mitochondria were stained with MitoTracker Green FM and MitoTracker Red CMXRos (#7514/#7512; Thermo Fisher Scientific, Waltham, MA, United States) (300 nM/ml; 250 nM/ml) for 30 min to detect their morphologies. Additionally, mitochondrial mass was also assessed with MitoTracker Green FM. Hoechst 33258 (1 μg/ml) was used to dye the nucleus for 5 min. Images were visualized by a confocal immunofluorescence microscope (Ti-E + A1R + STORM; Nikon, Tokyo, Japan). It must be noted that mitochondrial morphology was observed in myoblasts, since the density of mitochondria in myotubes was too high to distinguish. Mitochondrial morphology was analyzed with Mitochondria Analyzer (Chaudhry et al., 2020), a plugin of ImageJ software (1.5.2; National Institutes of Health, Bethesda, MD, United States). For each group, at least 100 mitochondria were detected and analyzed. The fluorescence intensity was measured by ImageJ software to determine the mitochondrial mass. For each group, four images were randomly selected and measured in × 200.

### Mitochondrial ROS and Mitochondrial Membrane Potential

Mitochondria were stained with JC-1 (HY-15534; MCE, NJ, United States) (10 μg/ml) and mitoSOX (M36008; Thermo Fisher Scientific, Waltham, MA, United States) (5 μM/ml) for 30 and 10 min, respectively, to detect their mitochondrial membrane potential (MMP) and mitochondrial ROS (mitoROS). Hoechst 33258 (1 μg/ml) was used to dye the nucleus for 5 min. Images were visualized by a confocal immunofluorescence microscope (Ti-E + A1R + STORM; Nikon, Tokyo, Japan). For JC-1, the fluorescence intensity of red and green was measured by ImageJ software (1.5.2; National Institutes of Health, Bethesda, MD, United States) to determine MMP. For each group, four images were randomly selected and measured in × 100. For mitoROS, the intensity was determined using a fluorescence spectrometer (Pro 200, Tecan, Männedorf, Switzerland) at excitation/emission wavelength of 510/580 nm.

### Statistical Analysis

*T*-test, one-way, and two-way ANOVA were used to determine the statistical significance of each group’s variance. Data are displayed as mean ± standard deviation, and *p* < 0.05 is considered as statistically significant.

## Results

### Demographic Data and Skeletal Muscle Evaluation of Patients With CAC

Analysis of demographic and clinical data revealed no difference in sex, age, height, and weight 6 months ago. The ratio of weight loss within the recent 6 months, BMI, biceps circumference, and waist circumference in CAC were significantly lower than in other two groups. Biochemical indicators of nutritional status such as albumin, prealbumin, and hemoglobin were decreased, while platelet was increased in CAC patients. There was no significant difference in the proportion of gastrointestinal tumors between CAC and NC. However, the tumor volume was much larger and clinical stage was more advanced in CAC patients (Table 1). These results demonstrated that the nutrition status was worsened and the burden of tumor was heavier in the cachetic than those without.

**TABLE 1:** Demographic and clinical characteristics among the groups.

	Control (*n* = 20)	NC (*n* = 27)	CAC (*n* = 29)
Age	58.71 ± 8.2	59.76 ± 10.02	58.67 ± 12.86
Male (%) Height (m)	77 1.65 ± 0.09	75.9 1.65 ± 0.08	77.8 1.68 ± 0.06
Body weight 6 months before diagnosis (kg) Body weight loss in 6 months at diagnosis (%)	66.8 ± 9.1 1.2 ± 0.3	65.5 ± 11.2 2.1 ± 1.0	65.9 ± 8.6 9.3 ± 3.5^a^
Body mass index	24.5 ± 3.12	24 ± 2.65	20.79 ± 1.99^a^
Biceps circumference (cm)	25.73 ± 1.92	25.16 ± 1.80	22.87 ± 1.74^a^
Waist circumference (cm)	85.68 ± 6.89	85.04 ± 7.39	80.65 ± 4.69^a^
Albumin (g/L)	42.98 ± 2.67	41.44 ± 3.2	36.57 ± 4.5^a^
Pre-albumin (g/L) Hemoglobin (g/L) Platelet (/L) Leukocyte (/L) Alanine transaminase (U/L) Aspartate transaminase (U/L) Tumor site Stomach and duodenum Colon and rectum Tumor volume (cm^3^) TNM stage (%) I II III IV	0.32 ± 0.06 135.8 ± 20.2 234 ± 83.2 6.5 ± 1.8 21 ± 7.3 18.9 ± 5.6	0.31 ± 0.09 132.62 ± 17.4 227 ± 64.4 6.4 ± 2.1 19.6 ± 11.2 20.3 ± 7.3 13 16 8.8 ± 9.2 9 (31.03) 8 (27.59) 10 (34.48) 2 (6.90)	0.23 ± 0.07^a^ 116.4 ± 29.2^a^ 266.5 ± 106.4^a^ 6.1 ± 2.0 12.5 ± 4 16.6 ± 3.7 14 14 49.2 ± 45.3^a^ 0 (0.00) 4 (14.81) 14 (51.85) 9 (33.33)

*^*a*^Represents *P* < 0.05 compared with the NC group.*

**FIGURE 1:**
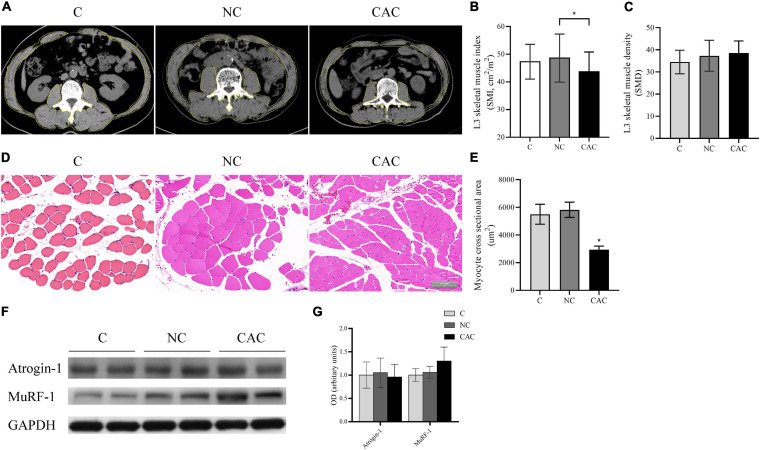
**(A)** Typical images of L3 vertebra from CT scan. Muscle content is outlined with a yellow line. **(B)** Quantification of SMI. **(C)** Quantification of SMD. **(D)** Typical HE staining images of fibers of rectus abdominis (*n* = 5 for NC and CAC groups; *n* = 3 for C group). **(E)** Quantification of CSA. **(F)** Levels of Atrogin-1 and MuRF-1 by Western blot. **(G)** Quantification of **(F)**. SMI, skeletal muscle index; SMD, skeletal muscle density; C, control, patients with benign disease; NC, patients of non-cachexia; CAC, cancer patients with cachexia. *p < 0.05. Data are displayed as mean ± standard deviation.

Furthermore, SMI indicated a reduction in CAC (CAC/NC/C = 43.3/48.7/47.3; *p* < 0.05) while SMD was unchanged (Figures 1A–C). Consistently, CSA also displayed a decline in CAC (Figures 1D,E). Since UPS is one of the main pathways responsible for muscle protein degradation (Baracos et al., 2018), we then examined its markers, MuRF-1 and Atrogin-1. Although an upregulated trend (*p* = 0.079) in the expression of MuRF-1 in CAC was observed, there was no significant difference among groups (Figures 1F,G). These results indicated a pronounced muscle loss among the cachetic patients enrolled in our study.

### Abnormal Mitochondrial Alterations and Activated Phosphorylated DRP1 in Cachetic Patients

**FIGURE 2:**
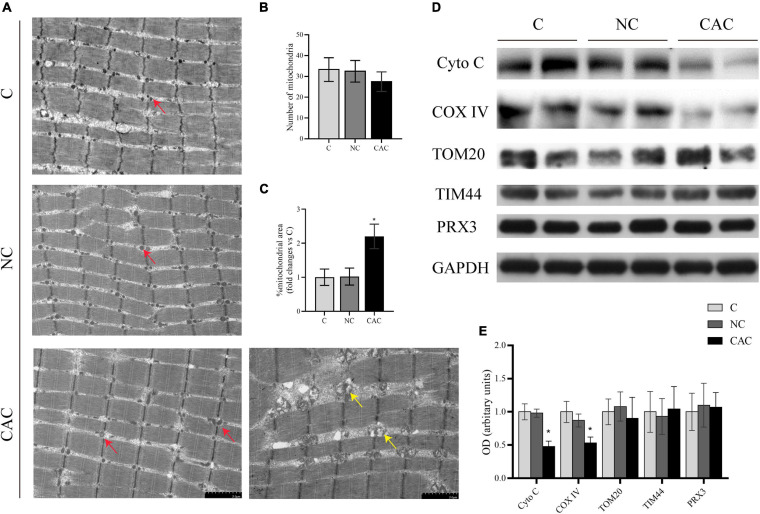
**(A)** Typical images under a transmission electron microscope. Red arrow indicates the normal mitochondrion, yellow arrow indicates the swollen and abnormal mitochondrion. **(B)** Quantification of mitochondrial number from the area of 96.9 μm^2^ (*n* = 3). **(C)** Quantification of mitochondrial area. **(D)** Levels of Cyto C, COX IV, TOM20, TIM44, and PRX3 by Western blot. **(E)** Quantification of **(D)** C, control, patients with benign disease; NC, patients of non-cachexia; CAC, cancer patients with cachexia. *p < 0.05. Data are displayed as mean ± standard deviation.

Observations from skeletal muscle showed that the area of mitochondria among myofibrils seemed to be smaller in NC and C. Moreover, in these mitochondria, the matrix also appeared deep and homogeneous, while in CAC, disorderly arranged and swollen mitochondria were frequently seen. The matrix was sparse (electron-lucent matrix) with a disarranged, abnormal cristae (Figure 2A). Indeed, the mitochondrial area was enlarged in CAC, compared to NC and C (CAC/NC/C = 1/1.02/2.15; *p* < 0.05), while mitochondrial number had a trend to be lower (CAC/NC/C = 27.5/32.6/33.25; CAC vs. C *p* = 0.08) (Figures 2B,C). In order to understand mitochondrial content and function more accurately, we further analyzed some markers, translocase of the outer membrane 20 (TOM20), translocase of the inner membrane 44 (TIM44), cytochrome c oxidase IV (COX IV), and cytochrome c (Cyto C) (Larsen et al., 2012; Alvarez-Paggi et al., 2017; Demishtein-Zohary and Azem, 2017; Ting et al., 2017). The results showed a suppressed COX IV and Cyto C in CAC compared with the other two groups, while TOM20 and TIM44 had no evident change (Figures 2D,E). Peroxiredoxin-3 (Prx3) is a mitochondrial protein involved in protecting radical-sensitive enzymes from oxidative damage, which could be used as a mitoROS marker (Lee et al., 2014; Poynton and Hampton, 2014). Among the three groups, no difference was seen in Prx3 level (Figures 2D,E). These data demonstrated abnormal alterations in mitochondrial structure in the atrophied muscle, accompanied with a change in mitochondrial function.

**FIGURE 3:**
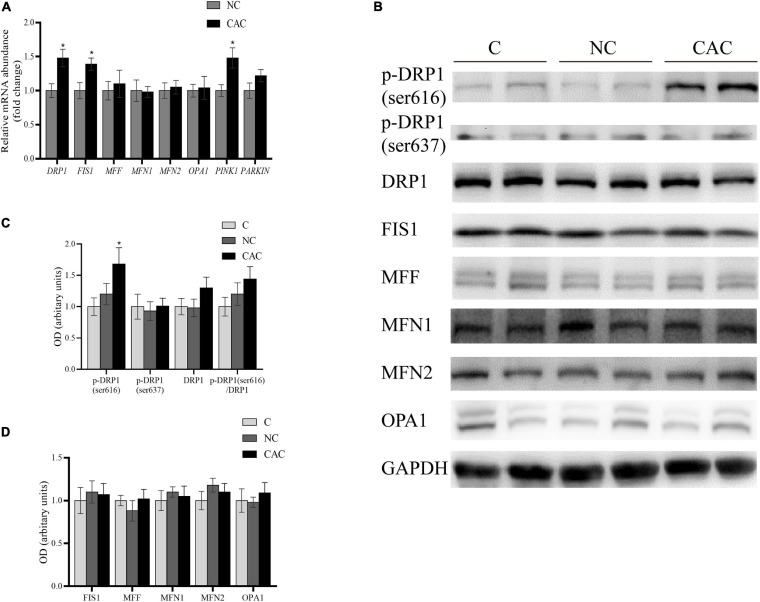
**(A)** Quantification of relative gene expressions of DRP1, FIS1, MFF, MFN1, MFN2, OPA1, PINK1, and PARKIN by real-time PCR. **(B)** Levels of DRP1, p-DRP1(ser616), p-DRP1(ser637), FIS1, MFF, MFN1, MFN2, OPA1, and GAPDH by Western blot. **(C)** Quantification of DRP1, p-DRP1(ser616), p-DRP1(ser637), and DRP1/p-DRP1 ratio. **(D)** Quantification of FIS1, MFF, MFN1, MFN2, and OPA1. C, control, patients with benign disease; NC, patients of non-cachexia; CAC, cancer patients with cachexia. *p < 0.05. Data are displayed as mean ± standard deviation.

Accumulating evidence has suggested that the morphologic modification in mitochondria could generate a significant impact on muscle content (Romanello et al., 2010; Touvier et al., 2015; Tezze et al., 2017; Favaro et al., 2019). Considering the noteworthy changes in mitochondrial structure, we examined the key proteins of the mitochondrial dynamic system. These factors included the fusion factors, MFN1, MFN2, and OPA1; fission factors, FIS1, MFF, and DPR1; and mitophagy factors, PINK1 and PARKIN. The results revealed that DRP1, FIS1, and PINK1 were enhanced at the mRNA level in CAC (Figure 3A). Further analysis for DRP1 and FIS1 did not exhibit a similar upregulation at the protein level. Alternatively, the phosphorylated state at the serine site, an essential posttranslational modification, is recognized as critical for the workout of DRP1. Phosphorylation of Ser616 in the GTPase effector domain (GED) stimulates DRP1 oligomerization and activates mitochondrial fission. Moreover, phosphorylation of Ser637 *via* protein kinase A (PKA) inhibits DRP1 activity, promoting a cytosolic localization and mitochondrial elongation (Taguchi et al., 2007; Rambold et al., 2011). We then analyzed their expressions and observed an upregulation at the ser616 site in CAC, and phosphorylation at the ser637 site remained unchanged (Figures 3B–D). These outcomes suggested an activation of DRP1, presenting probably mitochondrial fission condition in skeletal muscle of cachetic patients.

### The Role DRP1 Acts in a Cachexia Model *in vitro*

Lately, it was reported that the DRP1 level was crucial for mitochondrial function and muscle physiology (Romanello et al., 2010; Song et al., 2015; Favaro et al., 2019; Romanello and Sandri, 2021). Therefore, on the basis of clinical outcomes, we aimed to explore the relationship of DRP1, muscle loss, and mitochondrial function under a cachexia circumstance *in vitro*. We constructed a cachetic model by treating C2C12 myotubes with C26 CM, and then we used Mdivi-1, a specific DRP1 inhibitor, to block mitochondrial fission. Considering the possible suppressive effect on C2C12 myogenesis (Kim et al., 2013; Bloemberg and Quadrilatero, 2016), we firstly tested the toxicity of Mdivi-1 with different concentrations (1/5/10/20/30/40/50 μM). After differentiation, myotubes were treated with Mdivi-1 for 48 h, and the drugs did not have prominent influences on their diameters (Supplementary Figures 1A,B). Analysis of MyoD and MyoG, two growth markers of myoblasts, also did not imply any inhibition. Only when Mdivi-1 reached 40/50 μM was the expression of MyoG blocked, indicating certain toxic effects on myotubes in high concentrations (Supplementary Figures 1C,D).

In a preclinical model of cachexia, degeneration of mitochondrial function took place even in the early stage (Brown et al., 2017). To see whether this possible phenomenon existed in our model as well, we treated the myotube with CM for two time periods: 12 h (pre-cachexia) and 48 h (standard cachexia). Furthermore, the control and CM groups refer to the myotube cultured in DMEM of 2% HS and CM, respectively.

**FIGURE 4:**
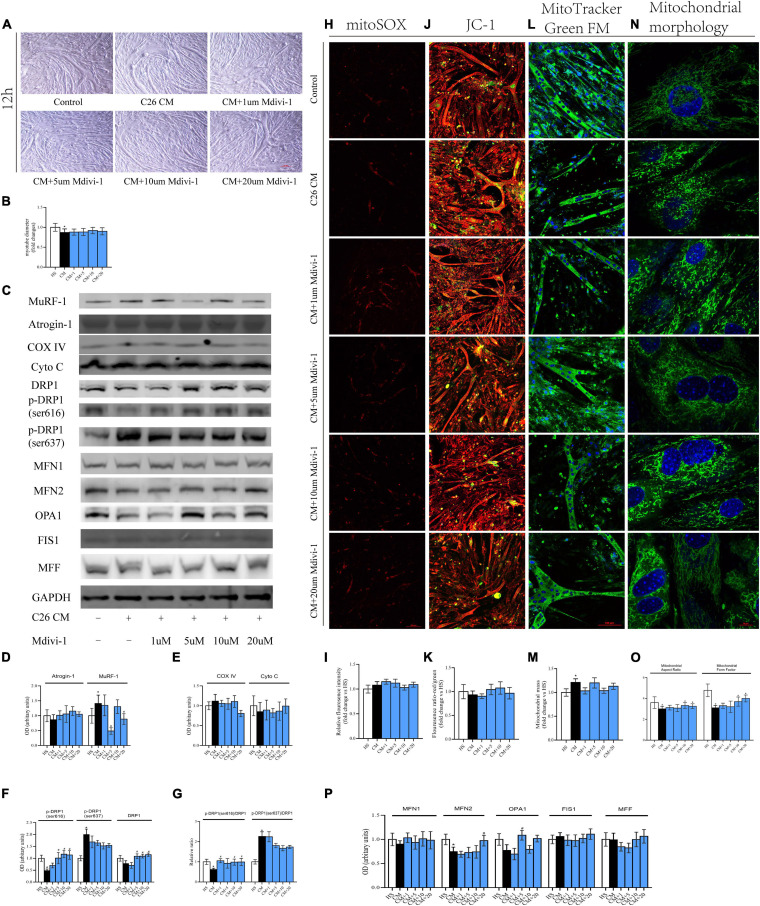
**(A)** Typical images of the C2C12 myotube cocultured with CM for 12 h. **(B)** Quantification of myotube diameter. **(C)** Levels of Atrogin-1, MuRF-1, DRP1, p-DRP1(ser616), p-DRP1(ser637), Cyto C, COX IV, and GAPDH by Western blot. **(D)** Quantification of Atrogin-1 and MuRF-1. **(E)** Quantification of Cyto C and COX IV. **(F)** Quantification of DRP1, p-DRP1(ser616), and p-DRP1(ser637). **(G)** Quantification of p-DRP1(ser616)/DRP1 and p-DRP1(ser637)/DRP1 ratio. **(H)** Typical images to analyze mitoROS by mitoSOX. **(I)** Quantification of mitoSOX with fluorescence ratio (Ex/Em = 510/580 nm). **(J)** Typical images to analyze MMP by JC-1. **(K)** Quantification of MMP with fluorescence intensity (red/green) ratio by ImageJ software. **(L)** Typical images to analyze mitochondrial mass by Mito-Tracker Green FM. **(M)** Quantification of mitochondrial mass with fluorescence intensity by ImageJ software. **(N)** Typical images to analyze mitochondrial morphology in myoblasts by MitoTracker Green FM. **(O)** Quantification of mitochondrial morphology by Mitochondrion Analyzer, a plugin of ImageJ software. FF (form factor: the reciprocal of circularity value) and AR (aspect ratio: major axis/minor axis of an ellipse equivalent to the object). **(P)** Quantification of MFN1, MFN2, OPA1, FIS1, and MFF; control: myotube cultured in DMEM of 2% HS; CM (conditional medium): myotube cultured in DMEM of 33% C26 conditional medium; *p < 0.05 vs. control group; #*p* < 0.05 vs. CM group. Data are displayed as mean ± standard deviation.

The results from 12 h showed that the myotube was thinner in CM compared with HS. Also, 10/20 μM Mdivi-1 was likely to mitigate this atrophy to some extent (Figures 4A,B). Cachexia circumstance induced the expression of MuRF-1, and this was inhibited by 5/20 μM Mdivi-1 (Figures 4C,D). However, we did not find a difference in COXIV, CytoC, mitoROS, and MMP among the groups (Figures 4C,E,H–K). Although the content of mitochondria was higher in CM, no significant change was observed after adding the drug (Figures 4L,M). For DRP1 and its phosphorylated levels, CM resulted in a reduction by 616 and an increase by 637, respectively. 5/10/20 μM Mdivi-1 attenuated the decline in 616, while 5/10/20 μM Mdivi-1 could also reverse this slight repression in DRP1 caused by CM (Figures 4C,F). The ratio of p-DRP1/DRP1 suggested a similar pattern as p-DRP1 (Figure 4G). In addition, analysis of other dynamic factors indicated that CM also induced a downregulation in fusion markers such as MFN2 and OPA, and some concentrations (20 μM for MFN2, 5 μM for OPA1) released these repressions (Figures 4C,P). Staining of mitochondrial morphology in myoblasts indicated that fission was dominant in CM, and 10/20 μM relieved this situation with an appearance of elongated mitochondria (Figures 4N,O). These phenomena demonstrated that certain concentrations of Mdivi-1 blunted the activity of DRP1 and atrogene MuRF-1, which was stimulated by cancer media in 12 h. However, in our study, no obvious alterations in mitochondrial function were observed.

**FIGURE 5:**
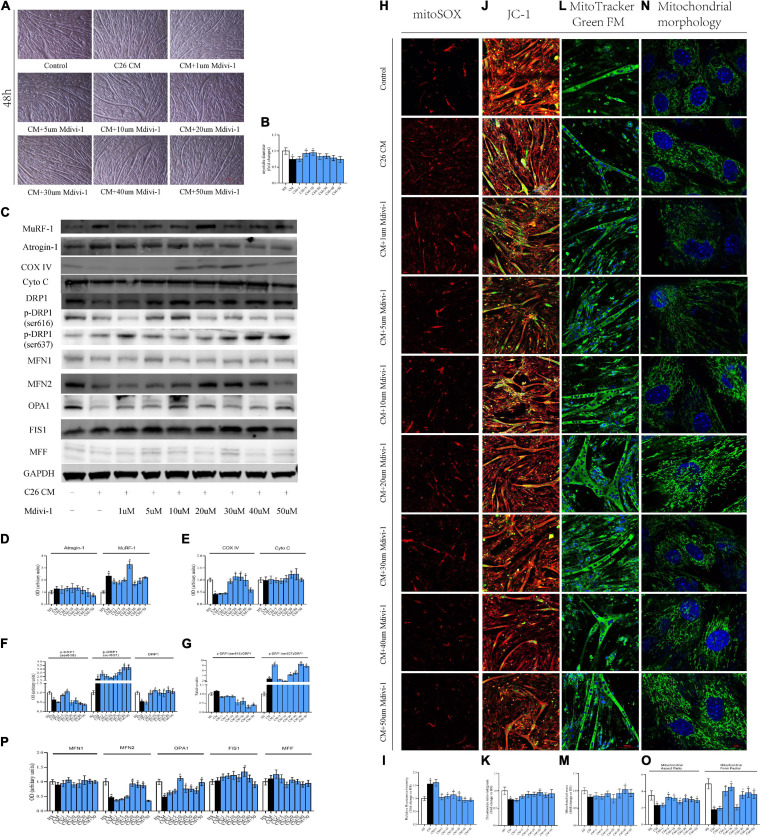
**(A)** Typical images of C2C12 myotube cocultured with CM for 48 h. **(B)** Quantification of myotube diameter. **(C)** Levels of Atrogin-1, MuRF-1, DRP1, p-DRP1(ser616), p-DRP1(ser637), Cyto C, COX IV, and GAPDH by Western blot. **(D)** Quantification of Atrogin-1 and MuRF-1. **(E)** Quantification of Cyto C and COX IV. **(F)** Quantification of DRP1, p-DRP1(ser616), and p-DRP1(ser637). **(G)** Quantification of p-DRP1(ser616)/DRP1 and p-DRP1(ser637)/DRP1 ratio. **(H)** Typical images to analyze mitoROS by mitoSOX. **(I)** Quantification of mitoSOX with fluorescence ratio (Ex/Em = 510/580 nm). **(J)** Typical images to analyze MMP by JC-1. **(K)** Quantification of MMP with fluorescence intensity (red/green) ratio by ImageJ software. **(L)** Typical images to analyze mitochondrial mass by Mito-Tracker Green FM. **(M)** Quantification of mitochondrial mass with fluorescence intensity by ImageJ software. **(N)** Typical images to analyze mitochondrial morphology in myoblasts by MitoTracker Green FM. **(O)** Quantification of mitochondrial morphology by Mitochondrion Analyzer, a plugin of ImageJ software. FF (form factor: the reciprocal of circularity value) and AR (aspect ratio: major axis/minor axis of an ellipse equivalent to the object). **(P)** Quantification of MFN1, MFN2, OPA1, FIS1, and MFF; control: myotube cultured in DMEM of 2% HS CM (conditional medium): myotube cultured in DMEM of 33% C26 conditional medium; *p < 0.05 vs. control group; #*p* < 0.05 vs. CM group Data are displayed as mean ± standard deviation.

Compared to 12 h, the atrophy for 48 h was more prominent, and 5/10 μM Mdivi-1 alleviated this muscle wasting. Interestingly, when Mdivi-1 was promoted to 30/40/50 μM, the anti-atrophy ability in myotubes began to fall. Moreover, myotubes in 50 μM were only marasmus as with CM (Figures 5A,B). Consistently, the upregulation of MuRF-1 induced in CM was also inhibited in 1/5/10/30 μM groups. Furthermore, we observed an increase trend in Atrogin-1 in CM, while this elevation could be gradually ameliorated by 30/40/50 μM Mdivi-1 (Figures 5C,D). Unlike 12 h, CM triggered a decrease in COX IV, which could be restored by 10/20/30/40 μM Mdivi-1 in this time period (Figures 5C,E). MitoROS production and MMP loss were seen in CM. Different concentrations (except for 1 μM) of Mdivi-1 effectively reduced mitoROS, and similarly, 5/10/20/30/40/50 μM mildly increased MMP, which reached statistical significance at 30 μM. Alternatively, Mdivi-1 of high concentrations was likely to improve the mitochondrial content, prominent for 40 μM (Figures 5H–M). As for the DRP1 series, CM induced a reduction in total DRP1, and 5/10/20/30/40/50 μM enhanced its expression. For phosphorylated sites, CM led to a workout pattern similar to 12 h. Moreover, accordingly, recovery was seen for 616 (5/10 μM) and 637 (30/40/50 μM) in some groups (Figures 5C,F). Consequences from p-DRP1/DRP1 revealed that Mdivi-1 decreased its level for 616, which was significant at 20/30/40/50 μM. In contrast, Mdivi-1 increased 637 expression, and this was evident at 20/30/40/50 μM (Figure 5G). For other fusion factors, CM generated a decline in MFN2 and OPA1, and different concentrations of Mdivi-1 partially ameliorated this effect (Figures 5C,P). Mitochondrial staining in myoblasts disclosed the activated fragmentation status in CM, but Mdivi-1 (from 10 to 50 μM) substantially changed this condition (Figures 5N,O). Since Mdivi-1 was reported to have a potential impact on complex I (Bordt et al., 2017), we also checked its expression to see the possible influence on myotube physiology. Our results did not indicate a significant difference in these groups. Besides, 40/50 μM Mdivi-1 produced an insignificant recovery in complex I, compared to CM (Supplementary Figures 1E,F). Taken together, these phenomena presented that the alterations induced by cachexia at 48 h were much more intense and extensive than at 12 h. Mdivi-1 mitigated the myotube atrophy *via* lessening the expression of atrogenes, MuRF-1, and Atrogin-1. It also moderately alleviated the suppressed mitochondrial function and ROS induced by CM. This was probably associated with its ability to reduce the activity of DRP1 and reverse the mitochondrial division.

**FIGURE 6:**
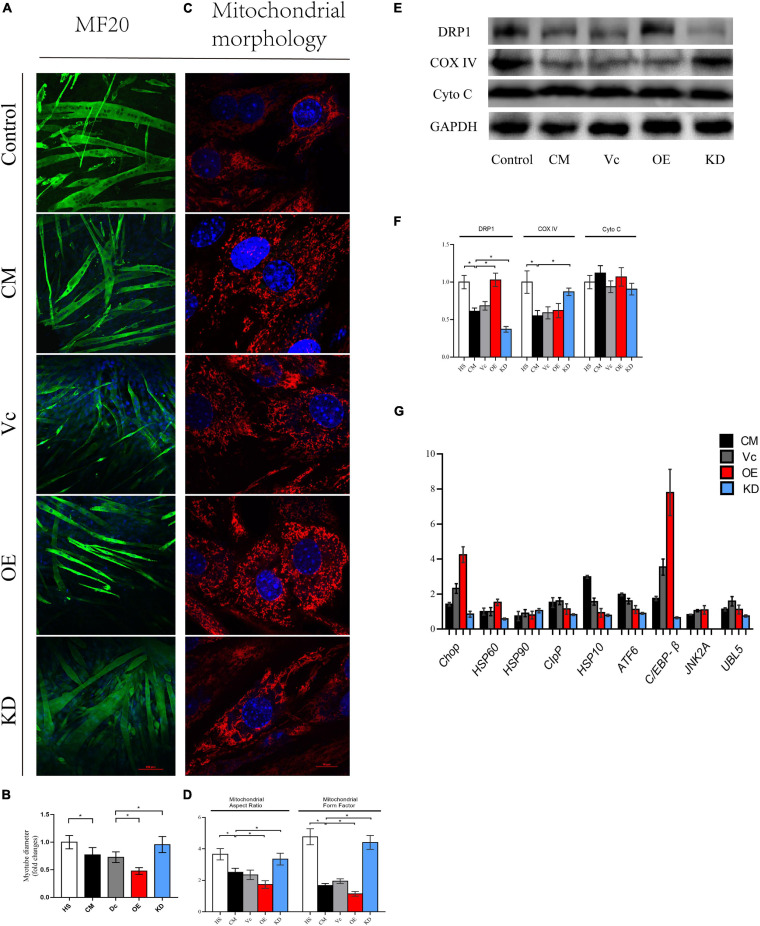
**(A)** Typical images of the C2C12 myotube cocultured with CM for 48 h. Myotube was stained with MF20. **(B)** Quantification of myotube diameter. **(C)** Typical images to analyze mitochondrial morphology by MitoTracker Red CMXRos. **(D)** Quantification of mitochondrial morphology by Mitochondrion Analyzer, a plugin of ImageJ software. FF (form factor: the reciprocal of circularity value) and AR (aspect ratio: major axis/minor axis of an ellipse equivalent to the object) **(E)** Level of DRP1, Cyto C, COX IV, and GAPDH by Western blot. **(F)** Quantification of DRP1, Cyto C, COX IV, and GAPDH. **(G)** Quantification of relative genes expression of *Chop*, *HSP60*, *HSP90*, *CIpP*, *HSP10*, *ATF6*, *C/EBP-*β, *JNK2A*, and *UBL5* by real-time PCR. *p < 0.05 vs. control group; #*p* < 0.05 vs. CM group. Data are displayed as mean ± standard deviation. Control: myotube cultured in DMEM of 2% HS CM: myotube cultured in control (2% HS), CM (myotube without infection in DMEM of 33% C26 conditional medium), Vc (myotube infected with control lentivirus in CM), OE (myotube infected with overexpressed lentivirus in CM), and KD (myotube infected with knockdown lentivirus in CM). Data are displayed as mean ± standard deviation.

To further understand the link between degradation of myotube and mitochondrial dynamics, we used lentiviruses to knock down and overexpress DRP1. The fluorescence of eGFP (>80%) implied that lentiviruses were successfully transduced into myoblasts (Supplementary Figure 2A). Of note, considering that excessive DRP1 knockdown might have a suppressive effect on differentiation of C2C12 myoblasts, we selected the cell line with a knockdown of 50–70%. Then, expression of DPR1 was confirmed for each vector (Supplementary Figures 2B,C). To our surprise, the myotube fusioned and differentiated smoothly in both DRP1 overexpression and knockdown groups in 3–4 days. Then, these myotubes were divided into five groups: control (2% HS), CM (myotube without infection in CM), Vc (myotube infected with control lentivirus in CM), OE (myotube infected with overexpressed lentivirus in CM), and KD (myotube infected with knockdown lentivirus in CM). The atrophy in Vc was similar in CM, and DRP1 knockdown mitigated this atrophy produced by cachexia while DRP1 overexpression exacerbated it (Figures 6A,B). Mitochondrial morphology revealed a noteworthy fragmentation in OE, while KD appeared to be fusion-dominant (Figures 6C,D). The expression of COX IV was found enhanced in KD, compared to CM and Vc, and we did not see a decrease in OE. Moreover, similar with results from Mdivi-1, DRP1 regulation did not alter the level of Cyto C (Figures 6E,F). Under stress conditions, when the degradation pathways are inadequate to repair the changes in mitochondrial translation or remove the accumulated oxidatively damaged proteins, a retrograde signal termed mitochondrial unfold protein response (mtUPR) is activated. Motivation of mtUPR improves protein folding, inhibits protein synthesis to alleviate ER stress, and eliminates damaged proteins (Callegari and Dennerlein, 2018). Since mitoROS production was noticeable in our model, we also analyzed some markers in mtUPR. Compared with control, CM, and Vc, both exhibited an upregulation at the mRNA levels of *Clpp*, *Hsp10*, *Atf6*, *C/ebp-*β, and *UBL5*. DRP1 overexpression promoted the levels of *Chop* (fourfolds), *Hsp60*, and *C/ebp-*β (eightfolds), while the overall level in DRP1 knockdown was low, especially for *Hsp90* and *Jnk2* (Figure 6G). These outcomes verified that downregulation of DRP1 set the disrupted mitochondrial dynamic back to balance, thus attenuating myotube atrophy and improving impaired mitochondrial function. Contrarily, upregulation of DRP1 intensified mitochondrial fission and subsequent atrophy.

## Discussion

Regarding demographic data, there are some points to be elucidated. All patients enrolled in our study had not received a systematic treatment, thus excluding other factors that might influence muscle mass. In this investigation, there were more male patients than women. This was in line with the gender prevalence of gastric and colorectal cancer (Brenner et al., 2014; Smyth et al., 2020), and males are also considered to be more susceptible to developing cachexia (Baracos et al., 2018). Additionally, as over 5% weight loss during the recent 6 months is usually used to screen the patients of cachexia, our results of SMI further confirmed that muscle contents in these patients were lower than those of weight-stable ones. This guaranteed the reliability of the study population in our research.

Myofibrillar protein is the main source of sarcomere construction and provides contractile function for skeletal muscle; the decrease in its content causes muscle atrophy and weakness. Degradation of myofibrillar proteins is dependent on ubiquitin proteasome and autophagy systems. Preclinical models of cachexia uncovered both of their activations (Baracos et al., 2018). However, in clinical patients, the conclusion is controversial. Zhang et al. (2020) found that both autophagy (LC3B/Beclin-1/P62) and UPS (MuRF-1/polyubiquitinylated protein) are activated in cachetic patients with gastrointestinal tumors. Some studies were partly in agreement with theirs, and elevated expressions of LC3B, ATG5, and ATG7 were seen (Johns et al., 2014; Aversa et al., 2016; de Castro et al., 2019). In contrast, some other clinical research studies did not discover motivations in these systems, regardless of gastrointestinal tumors (D’Orlando et al., 2014; Taskin et al., 2014; MacDonald et al., 2015; Stephens et al., 2015) or lung tumors (Op den Kamp et al., 2013). Our results matched with the latter conclusion, even though there was an upward trend in MuRF-1.

On the other hand, probes in animal indicated conspicuous disorders and abnormalities in mitochondria during CAC and sarcopenia (Tzika et al., 2013; Leduc-Gaudet et al., 2015; Barreto et al., 2016; Chalmers et al., 2016; Brown et al., 2017), but few were reported in clinical areas. In the rectus abdominis of sarcopenia and CAC, Zhang et al. found that sarcomeres and Z-lines were misaligned, and vesicle and autophagosome were formed in some severely disrupted sarcomere areas. Mitochondria were swollen and characterized by an absence of cristae (Zhang et al., 2020). Likewise, de Castro et al. (2019) discovered that triads were disrupted and mitochondrial morphology was altered in gastrointestinal cachetic patients. What is more, they noticed that the mitochondrial area was increased but the mitochondrial DNA (mtDNA) copy number had not changed (de Castro et al., 2019). Our results were partly consistent with these previous studies. Swollen and enlarged mitochondria are considered as a symbol of dysfunction and unable to perform normal autophagy and fusion activities (Narendra et al., 2008; Navratil et al., 2008). It is also associated with loss of adenosine-5′-triphosphate (ATP) content, which leads to a decline in oxidative phosphorylation (OXPHOS) (Gupta et al., 2010). Thus, these observations probably suggested an aberrant change in mitochondrial physiology. In our study, mitochondrial number was not statistically changed. Even though a trend of downregulation was detected, this could be explained by a reduction in muscle mass. Actually, it is believed that loss of mitochondria occurs in the late stage of cachexia, while mitochondrial degeneration takes place at the early stage (Brown et al., 2017).

TOM20 and TIM44 are mitochondrial receptor complexes located at the outer and inner mitochondrial membranes, respectively. Furthermore, they are involved in the translocation of cytosolically mitochondrial pre-protein, while COX IV and Cyto C participate in mitochondrial respiration (Larsen et al., 2012; Alvarez-Paggi et al., 2017; Demishtein-Zohary and Azem, 2017; Ting et al., 2017). In our study, we found that COX IV and Cyto C were reduced and TOM20 and TIM44 remained unaltered. Since we did not examine the respiratory chain, it was uncertain whether there was an impairment. On this field, correlated studies were also few. In research carried out in patients with lung cancer, Op den Kamp et al. (2015) did not find noteworthy changes in OXPHOS expressions between pre-cachexia and cachexia. Therefore, there was also a possibility that our reduction was induced by mitochondrial content.

Excessive ROS has been indicated in some preclinical and clinical studies of cachexia (Puig-Vilanova et al., 2015; Brown et al., 2017; Chacon-Cabrera et al., 2017). However, as the main source of ROS, mitochondria had not been investigated. We did not find a difference in Prx3, a novel marker of mitoROS. However, it is still worth noting here that its oxidation state may better represent mitoROS, which we did not perform successfully in native electrophoresis. Moreover, the exact level remains to be explored.

Further, we saw an activation of phosphorylated DRP1 at the serine 616 site in cachexia. Since DRP1 is the major regulator of fission, this may imply an enhanced mitochondrial fragmentation in atrophied skeletal muscle. Similar events had also occurred in several clinical studies. Marzetti et al. (2017) noticed that Fis1 transcription was significantly increased in elderly patients with gastric cancer cachexia. This also appeared in the study from de Castro et al. (2019). Mitochondrial fission is an important mechanism to ensure natural operation by transferring dysfunctional mitochondria to mitophagy (Twig et al., 2008; Dagda et al., 2009). In our study, it was assumed that this boost might be attributed to the accumulation of dysfunctional mitochondria we visualized in TEM, which also was consistent with our results in mitophagy.

Meanwhile, this phenomenon in clinical was quite different from the condition in preclinical studies. Analysis of literature and the four genome-wide transcriptome datasets of cancer-induced cachexia mouse models has drawn another conclusion (van der Ende et al., 2018). DRP1/Oma1 and Mfn2/Opa1, involved in promoting fission and fusion, respectively, were consistently reported to be downregulated. At the same time, most other genes of fission and fusion did not change. Although some conflicting outcomes existed [such as Mfn2 (Fontes-Oliveira et al., 2013), Fis1 (White et al., 2012; Brown et al., 2017)], these evidences as a whole provided support for the hypothesis that mitochondrial dynamics are impaired in mouse model.

However, in cachexia, results from the clinical trial have been impinging the outcomes at the preclinical trial setting (Tomasin et al., 2019; Martin and Freyssenet, 2021). It may be due to the heterogeneity and complexity in tumor types and individuals. Besides, this also explains the “lack in translation” of preclinical-to-clinical. There are more than a hundred clinical trials being conducted, but none of them transformed to an approved drug, including the recent failure of anamorelin and enobosarm in a Phase III clinical trial (Le-Rademacher et al., 2017). Moreover, this serves to further increase caution to improve the predictive power for preclinical models.

Here, one thing is noteworthy during the process of our study *in vitro*. Initially, in our supposition, the fission machinery would be activated in cell models similar to clinical. Therefore, we used the antagonist of DRP1, which probably has the potential to reverse the atrophy. To our surprise, from the levels of phosphorylated 616 and 637 sites, it could be inferred that the activity of DRP1 was decreased in CM compared to HS. This matched with the major conclusion in preclinical experiments. However, mitochondrial staining revealed that mitochondrial fragmentation was dominant in cachexia. Moreover, importantly as well as strikingly, inhibition of division could attenuate muscle atrophy. These conflicting phenomena challenged our knowledge and ability to explain.

In physiological conditions, fusion and fission machineries are balanced. Overactivation or repression in either system would disrupt the equilibrium and thus generate subsequent impairment to mitochondria and cell health in muscle. In some conditions, the impairment could be reversed when this balance is restored, even if levels of fission and fusion have been changed. Chen et al. (2015) found that MFF-deficient mice developed severe dilated cardiomyopathy, with the decreased myocardial mitochondrial density, respiratory chain function, and excessive mitochondrial autophagy. Yet, simultaneous knockout of MFN1 rescued the concerning cardiac dysfunction, prolonged life span, and improved respiratory chain function (Chen et al., 2015). Romanello et al. (2019) uncovered that deletion of OPA1 in mice caused malignant symptoms such as muscle atrophy, systemic inflammatory response, precocious epithelial senescence, and premature death induced by the activation of FGF21. Simultaneous deletions of DRP1 and OPA1 would result in blunted autophagy and mitophagy, leading to accumulation of abnormal mitochondria, and eventually leading to ER stress, muscle atrophy, and weakness. However, the double knockouts reduced oxidative stress, denervation, and inflammation caused by FGF21 and rescued the lethal phenotype caused by OPA1 single deletion (Romanello et al., 2019). Song et al. (2017) revealed that mice with simultaneous deletions of DRP1 and MFN1/MFN2 in the heart had alleviated symptoms of cardiomyopathy and survived longer than those with merely deletion of DRP1 or MFN1/MFN2. These studies clearly demonstrated that the new balance acquired from inhibition of both fusion and fission machineries is more beneficial than the imbalance caused by the inhibition or activation of one machinery alone.

**FIGURE 7:**
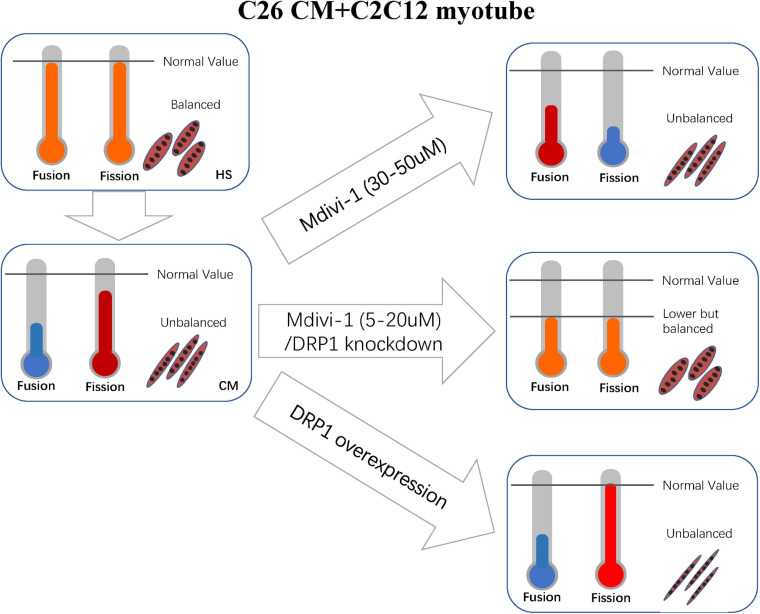
Correlated machinery elucidated in section “Discussion.”

Synthesizing the above conditions, the reasonable explanation is that the repression of fusion was even sharper than fission (Figure 7). Our consequences of other suppressed fusion proteins support this explanation as well. Apart from this, we also discover some clues favoring our argument. A review by van der Ende et al. (2018) suggested that the literatures with suppressed expressions of MFN1, MFN2, and OPA1 were more than those of DPR1 and FIS1 in preclinical cachexia.

Altogether, *in vitro* the inhibition of division at the concentration of 5–10 (a mild working concentration), along with the light effect on increasing fusion, transferred mitochondrial dynamics into a new balance. Consequently, these mechanisms restored mitochondrial function and atrophy. For 20 μM, the myotube is not recovered as expected, and atrogenes such as MuRF-1 also appeared to increase. We could not fully explain this phenomenon, but this may be due to a rebound reaction when the new balance is broken again. In the concentrations of 30–50 μM, myotube atrophy is prominent, and this might be caused by the disruption of new balance. Mdivi-1 was reported to a possible block on complex I when its concentration was over 25 μM (Bordt et al., 2017). However, this was not confirmed in myoblast. Thus, we checked this marker and observed a slight decrease in complex I in CM. Then we found a mild recovery in high concentrations of Mdivi-1 instead of the reduction we initially supposed. This was perhaps a result of the reduced functions of respiration in CM, as Mdivi-1 had an effect on ameliorating this condition. One step further, knockout and overexpression of DRP1 certified our arguments. The balance between fusion and fission is delicate and intricate. Although DRP1 is crucial in regulating division, other factors such as FIS1 and MFF also participate in the process. Considering the self-regulating compensatory mechanism of cells, more in-depth research studies are required to obtain a more precise understanding in this field.

In conclusion, our investigation in atrophied skeletal muscle from cachetic patients disclosed some abnormal alterations in mitochondria, including enlarged area, aberrant morphology, and partially functional change. Meanwhile, upregulated phosphorylation of DRP1 in this population suggested an active fragmentation in mitochondria. *In vitro*, we used the specific DRP1 inhibitor, Mdivi-1, to regulate the balance of mitochondrial dynamics and found that muscle atrophy was ameliorated after adding this drug. This was associated with its effect on mitigating degradation of muscle protein, restoring mitochondrial function and MMP, and reducing mitoROS. Knockdown and overexpression of DPR1 further verified the significance of mitochondrial balance in myotube. These phenomena shed new light on the importance of mitochondrial dynamics in cachetic atrophy, which was dominated by DRP1. The corresponding targets may provide reference for future therapy.

## Data Availability Statement

The datasets presented in the study are now publicly available in Figshare, and the link can be found below: doi: https://doi.org/10.6084/m9.figshare.15040689.v1.

## Ethics Statement

The studies involving human participants were reviewed and approved by Ethics Committee of Zhongshan Hospital. The patients/participants provided their written informed consent to participate in this study. Written informed consent was obtained from the individual(s) for the publication of any potentially identifiable images or data included in this article.

## Author Contributions

GW and QM: conception and design. XM, YG, and QM: development of methodology. QM, XM, XS, and LS: acquisition of data (including collection of clinical samples and patients’ information, etc.). QM, JH, and HW: analysis and interpretation of data (e.g., statistical analysis, biostatistics, and computational analysis). XM, YG, QM, and GW: writing, review, and/or revision of the manuscript. QZ, QX, and YG: administrative, technical, or material support. GW: study supervision. All authors contributed to the article and approved the submitted version.

## Conflict of Interest

The authors declare that the research was conducted in the absence of any commercial or financial relationships that could be construed as a potential conflict of interest.

## Publisher’s Note

All claims expressed in this article are solely those of the authors and do not necessarily represent those of their affiliated organizations, or those of the publisher, the editors and the reviewers. Any product that may be evaluated in this article, or claim that may be made by its manufacturer, is not guaranteed or endorsed by the publisher.

## Supplementary Material

Supplementary Figure 1 **(A)** Typical images of C2C12 myotube co-cultured with CM for 48 h. **(B)** Quantification of myotube diameter. **(C)** Level of MyoD and MyoG by western blot. **(D)** Quantification of MyoD and MyoG #*P* < 0.05 vs. HS group **(E)** Level of MyoD and MyoG by western blot. **(F)** Quantification of complex I ^∗^*P* < 0.05 Control: myotube cultured in DMEM of 2% HS CM (conditional medium): myotube cultured in DMEM of 33% C26 conditional medium; ^∗^*P* < 0.05 vs. Control group; #*P* < 0.05 vs. CM group Data are displayed as mean ± standard deviation.

Supplementary Figure 2 **(A)** Typical images to analyze fluorescence intensity of eGFP in myoblast. **(B)** Level of DRP1, GAPDH by western blot. **(C)** Quantification of **(B)** ^∗^*P* < 0.05 Vc (myoblast infected with control lentivirus), OE (myoblast infected with overexpressed lentivirus), KD (myoblast infected with knockdown lentivirus). Control: myotube cultured in DMEM of 2% HS CM (conditional medium): myotube cultured in DMEM of 33% C26 conditional medium; ^∗^*P* < 0.05 vs. Control group; #*P* < 0.05 vs. CM group Data are displayed as mean ± standard deviation.
