# Supplementary figures and images for: Phosphorylation of Dynamin-Related Protein 1 (DRP1) Regulates Mitochondrial Dynamics and Skeletal Muscle Wasting in Cancer Cachexia
Source: Front Cell Dev Biol. 2021 Aug 5;9:673618. doi: 10.3389/fcell.2021.673618 (PMC8375307; doi:10.3389/fcell.2021.673618)

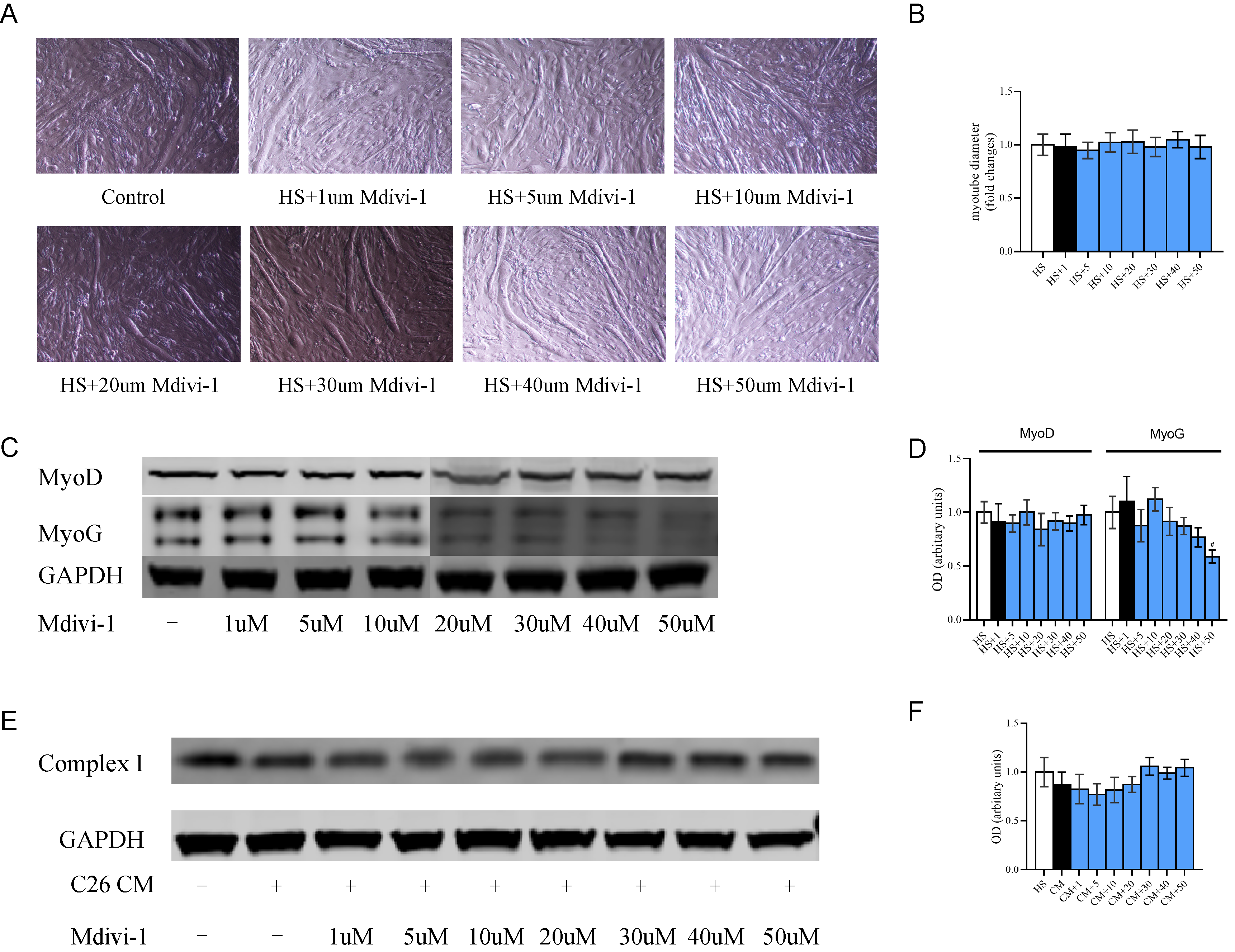

Supplement: Supplementary Figure 1 — (A) Typical images of C2C12 myotube co-cultured with CM for 48 h. (B) Quantification of myotube diameter. (C) Level of MyoD and MyoG by western blot. (D) Quantification of MyoD and MyoG #P < 0.05 vs. HS group (E) Level of MyoD and MyoG by western blot. (F) Quantification of complex I ∗P < 0.05 Control: myotube cultured in DMEM of 2% HS CM (conditional medium): myotube cultured in DMEM of 33% C26 conditional medium; ∗P < 0.05 vs. Control group; #P < 0.05 vs. CM group Data are displayed as mean ± standard deviation. [file Image_1.tif]

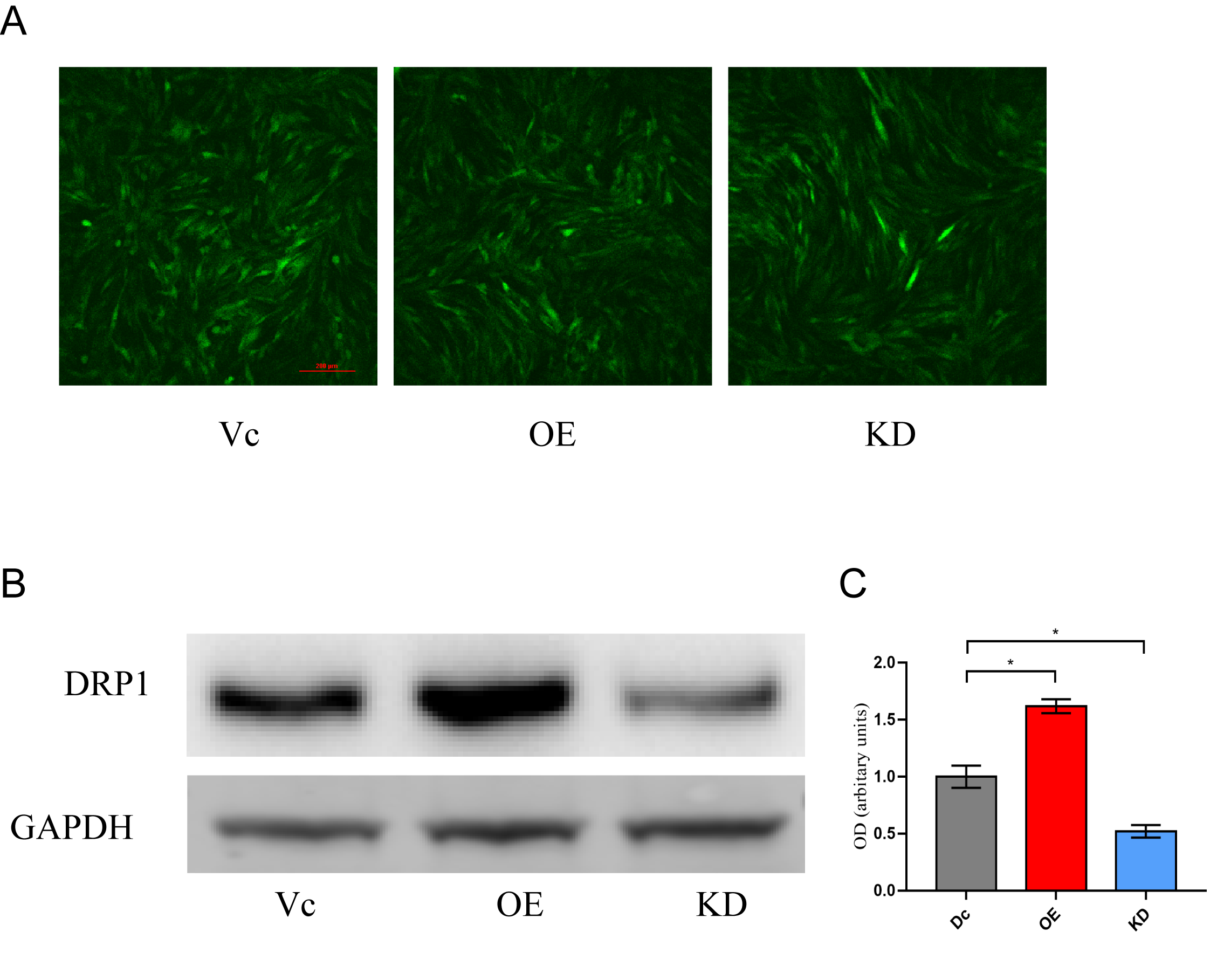

Supplement: Supplementary Figure 2 — (A) Typical images to analyze fluorescence intensity of eGFP in myoblast. (B) Level of DRP1, GAPDH by western blot. (C) Quantification of (B) ∗P < 0.05 Vc (myoblast infected with control lentivirus), OE (myoblast infected with overexpressed lentivirus), KD (myoblast infected with knockdown lentivirus). Control: myotube cultured in DMEM of 2% HS CM (conditional medium): myotube cultured in DMEM of 33% C26 conditional medium; ∗P < 0.05 vs. Control group; #P < 0.05 vs. CM group Data are displayed as mean ± standard deviation. [file Image_2.TIF]
