# Supplementary material for: Phosphorylation of Dynamin-Related Protein 1 (DRP1) Regulates Mitochondrial Dynamics and Skeletal Muscle Wasting in Cancer Cachexia
Source: Front Cell Dev Biol. 2021 Aug 5;9:673618. doi: 10.3389/fcell.2021.673618 (PMC8375307; doi:10.3389/fcell.2021.673618)
Supplement: Supplementary file 3 [file Table_1.DOCX]

**Table S1 Primer sequence**

| **gene** | **Primer-F** | **Primer-R** |
| --- | --- | --- |
| Fis1 | GTAAAGGCATCGTGCTGCTC | ACGGCCAGGTAGAAGACGTA |
| DRP1 | CTCTGGCCAATAGAAATGGAACA | TCATCCACGGGTTCACCGTA |
| Mff | GCGAATGAGGGTCCCAGAAA | TGTTTTCAGTGCCAGGGGTT |
| Mfn1 | GTTGGAGCGGAGACTTAGCA | TCCGAGATAGCACCTCACCA |
| Mfn2 | AGAACTGGACCCCGTTACCA | GAAGCAATTGGTGGTGTGGC |
| Opa1 | GGAAGTCCATGCGCCATT | CACTAAAGACTGGCAGACCTC |
| Pink1 | CGGACGCTGTTCCTCGTTAT | AAATCTGCGATCACCAGCCA |
| Parkin | ATTTAACCCAGGAGAGCCGC | AGCTGGAAGATGCTGGTGTC |
| GAPDH | GGGAAGCCCATCACCATCTT | TCGTGGTTCACACCCATCAC |
| UBL5 | TCGGAAAGAAAGTCCGCGTT | GAACGATCTTGTTCCAGCGG |
| JNK2 | AGGTGGCGGACTCAACTTTC | CGAGTTCACGGTAGGCTCTC |
| C/EBPβ | GACAAGCTGAGCGACGAGTA | TGCTTGAACAAGTTCCGCAG |
| ATF6 | GAACTTCGAGGCTGGGTTCA | CTTGCAGCTCACTCCCAGAA |
| HSP10 | CTCCCAGAATATGGAGGCACC | TCAGTCGACATACTTTCCAAGAA |
| ClpP | CACACCAAGCAGAGCCTACA | CTCCTTAGGTGCTTGTCGGG |
| HSP60 | AGTCCTTCGCCAGATGAGAC | TGCAACAGTGACCCCATCTTT |
| HSP90AB1 | GACTGGGAAGACCACTTGGC | TCTCAAAAAGGTCAAAGGGAGC |
| CHOP | CCTTTCACCTTGGAGACGGT | TCTGTCAGCCAAGCTAGGGA |
| HSP90 | CCACCACCCTGCTCTGTACTA | CCTCTCCATGGTGCACTTCC |
| Dnm1l | GAGGATCCCCGGGTACCGGTCGCCACCATGGAGGCGCTGATCCCGGTCATC | TCCTTGTAGTCCATACCCCAAAGATGAGTCTCTCGGATTTC |
